# Biophysical potential of crop residues for biochar carbon sequestration, and co‐benefits, in Uganda

**DOI:** 10.1002/eap.1984

**Published:** 2019-08-30

**Authors:** Dries Roobroeck, Rebecca Hood‐Nowotny, Dianah Nakubulwa, John‐Baptist Tumuhairwe, Majaliwa Jackson Gilbert Mwanjalolo, Isaac Ndawula, Bernard Vanlauwe

**Affiliations:** ^1^ International Institute of Tropical Agriculture Natural Resource Management Unit, c/o ICIPE KISE Road, P.O. Box 30772‐00100 Nairobi Kenya; ^2^ Institute of Soil Research University of Natural Resources and Life Sciences Peter‐Jordan‐Straße 82 Vienna 1190 Austria; ^3^ Department of Agricultural Production Makerere University University Road, P.O. Box 7062 Kampala Uganda; ^4^ Department of Geography, Geo Informatics and Climatic Sciences Makerere University University Road, P.O. Box 7062 Kampala Uganda

**Keywords:** biomass pyrolysis, climate change mitigation, low carbon energy, natural resource management, prospective modeling, scenario analysis, soil carbon, tropical agro‐ecosystem

## Abstract

Increasing organic matter/carbon contents of soils is one option proposed to offset climate change inducing greenhouse gas (GHG) emissions, under the auspices of the UNFCC Paris Agreement. One of the complementary practices to sequester carbon in soils on decadal time scales is amending it with biochar, a carbon rich byproduct of biomass gasification. In sub‐Saharan Africa (SSA), there is a widespread and close interplay of agrarian‐based economies and the use of biomass for fuel, which makes the co‐benefits of biochar production for agriculture and energy supply explicitly different from the rest of the world. To date, the quantities of residues available from staple crops for biochar production, and their potential for carbon sequestration in farming systems of SSA have not been comprehensively investigated. We assessed the productivity and usage of biomass waste from maize, sorghum, rice, millet, and groundnut crops; specifically quantifying straw, shanks, chaff, and shells, based on measurements from multiple farmer fields and household surveys in eastern Uganda. Moreover, allometric models were tested, using grain productivity, plant height, and density as predictors. These models enable rapid and low‐cost assessment of the potential availability of feedstocks at various spatial scales: individual cropland, farm enterprise, region, and country. Ultimately, we modeled the carbon balance in soils of major cropping systems when amended with biochar from biomass residues, and up‐scaled this for basic scenario analysis. This interdisciplinary approach showcases that there is significant biophysical potential for soil carbon sequestration in farming systems of Uganda through amendment of biochar derived from unused residues of cereals and legume crops. Furthermore, information about these biomass waste flows is used for estimating the rates of biochar input that could be made to farmlands, as well as the amounts of energy that could be produced with gasifier appliances.

## Introduction

Biochar by definition is the organic end product of a pyrolysis process (i.e., thermal decomposition in a low oxygen atmosphere), which is intended specifically for soil amendment. Microbial decomposition of biochar is slow and dependent on the biomass feedstock and pyrolysis temperature; consequently, adding biochar to soil can lock carbon (C) away for several years to centuries (Wang et al. 2016). In experiments with maize–soybean rotations in Kenya, amended with acacia wood char at a rate of 28 kg C/ha, results suggested that 60% of the carbon remained in the top 20 cm soil over a decade, with part of the loss attributable to erosion (Katterer et al. 2019). Other studies with biochar, made from various types of residues, have demonstrated similar or lower rates of carbon retention in soils under intensive crop production (Häring et al. 2017, de la Rosa et al. 2018). In another Kenyan study, the input of charcoal led to a 27% decrease in carbon dioxide (CO_2_) emissions from maize croplands that had been converted to agriculture several decades prior, and was argued to be a result of enhanced stabilization of plant‐derived C (Kimetu and Lehmann 2010). Generally, it is suggested that the inherent stability of pyrogenic organic matter negates its requirement for protection from aggregates and/or clay minerals to be stable, implying that biochar can accumulate higher concentrations of C before a saturation point is reached, in contrast to manure or straw (Smith 2016). Moreover, the addition of pyrolyzed carbon to soil also does not appear to induce significant microbial immobilization of nitrogen (N) in contrast to “raw” carbon, which has massive implications for maintaining crop yields (Hood‐Nowotny et al. 2018).

In sub‐tropical and tropical agroecosystems, meta‐analysis has shown that biochar addition to soils can increase crop yields, on average, by 25%, in stark contrast to temperate regions where observed responses are negligible or slightly negative (Jeffery et al. 2017). This was clearly demonstrated by persistent grain yield increases of maize–soybean rotations in Kenya over 10 yr, without and with minimal inorganic fertilizer input to cereal phases, following a single application of charcoal at the start of the experiment; where mean yield responses amounted to between 0.9–1.3 and 0.3–0.8 Mg/ha for the maize and soybean crop, respectively (Katterer et al. 2019). Responses of grain and residue yields from crops to soil biochar amendment are generally found to decrease with increasing rates of input, and applications as little as 0.5 Mg DM/ha (DM, dry mass) have shown significant positive effects on crop productivity (Liu et al. 2013). The observed gains in crop production likely result from the multiple beneficial impacts of biochar amendment on soil properties, such as the exchange capacity, aggregation, and hydraulic conductivity (Liang et al. 2006, Blanco‐Canqui 2017), as well as the poor nutritional status of farmland soils that were studied; which is a widespread phenomenon across SSA.

When organic material is pyrolyzed in a gasifier system and is carbonized, it releases heat and synthetic combustible gases, which can contribute to effectively reducing wood consumption and GHG emissions per unit energy compared to biomass incineration systems (Sanford and Burney 2015). The advantage of pyrolysis‐based technologies is that they allow substitution of wood‐based fuels with crop residues of low energy density, e.g., straw, chaff, husks, shell, or pruning from crops. Multiple biomass gasification appliances are on the market for households and businesses, offering direct energy savings or income generation. As a rule, the more heat and synthetic gas that is drawn from a feedstock, the less char remains, and the ratios are mainly determined by pyrolysis temperatures, airflows, and feedstock types. Utilization of renewable biomass from crop residues for gasifier energy appliances in unison with soil biochar C sequestration could readily mitigate GHG emissions in farming systems of SSA, while increasing access to energy for households and businesses.

Large numbers of farmers in SSA depend on biomass resources for agriculture and energy, which makes the prospective for biomass gasification systems and biochar amendment to soils very different as compared to those in fully developed economies, which have been previously investigated (e.g., Bach et al. 2016, Amundson and Biardeau 2018). The additional income that could be earned by generating power from waste biomass, as well as increasing crop yields through applications of biochar in farming systems of SSA, could create ample incentives to sequester C in soils for climate mitigation. How much of GHG emissions could be offset via biochar inputs to soils is hotly debated, and relies on the availability of biomass wastes, pyrolytic conditions, and decomposition rates (Schlesinger and Amundson 2018). Residues from staple food crops are an obvious and promising choice for bioenergy and biochar production from a sustainability perspective. Because farmers utilize agricultural wastes for a variety of purposes, like animal fodder, soil surface cover, and construction, not all of it is realistically available for biochar. Therefore, it is critical that we factor in the allocation of biomass resources to estimate how much can be diverted to biochar C sequestration and other benefits in farming systems.

This study assesses the biophysical potential to sequester C in soils via biochar derived from by‐product residues of maize, sorghum, rice, millet and groundnut crops in smallholder farming enterprises of eastern Uganda, separately quantifying straw and non‐straw fractions (i.e., shanks, chaff, and shells). In so doing, a model‐based framework was compiled to determine the amount of C that could be sequestered in soils through biochar made from crop biomass wastes under varying scenarios of competition and decomposition, at both farmer field as well as national production scale. We also derive information about the corresponding rates of biochar amendment to farmlands and quantities of synthetic gas that can be generated for investigating the viability of implementing the technology in agricultural and energy systems of Uganda.

## Methods

### Description study area

**Figure 1:**
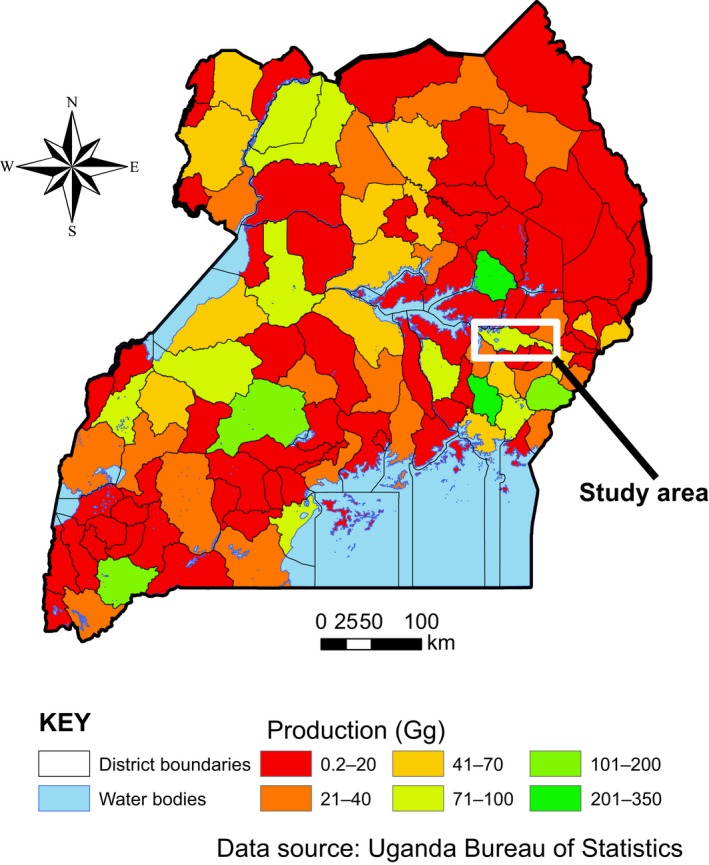
Map of Uganda showing the total grain production levels of maize, sorghum, rice, millet, and groundnut for individual districts, and the location of our study area.

Research activities took place over an area of approximately 250 km² in the Lake Kyoga basin situated in eastern Uganda; between 0°45′ N and 1°05′ N, and 33°47′ E and 34°05′ E (Fig. 1). Altitudes in the study area range from 910 to 1220 m above sea level and the climate is sub‐humid, with bimodal precipitation of 900–500 mm and mean annual temperature of 32.5°C. The topography of the landscape is undulating, and pronounced gradients in soil properties are found between individual farmer fields (Appendix S1: Table S1), which may lead to substantial variation of attainable crop yields. Production of cereal and legume crops is the main economic activity in the study area, generating approximately 3% of all maize, millet, sorghum, rice, and groundnut in the country (Fig. 1). Agro‐ecological conditions, rates of crop productivity and typologies of farming systems in the study area are representative for other mid‐altitude highland regions in the country.

### Characterization of cropping systems and residue allocation

Farmer associations operating within the study area were contacted at the start of the research project, and they delegated 27 female and 33 male members to attend a 1‐d workshop. Discussions with six member focus groups and individual surveys of all 60 farmers were held to identify the major staple crops being grown (Table 1), and select the five crops that were studied. The focus group discussions also led to distinguishing four major classes of biomass usage; fodder, construction, mulching (i.e., soil cover or incorporation), and cooking fuel. Individual surveys were taken during home visits and included semi‐structured questions about (1) household composition, education, and sources of income; (2) total area of land and livestock owned by the household; (3) crops cultivated and fertilizer investments over the past three years; and (4) whether or not they allocate individual crop residues to the four major usages. Information provided by farmers was verified by research staff at the homestead after completing surveys. The proportion of respondents that did not indicate using a residue for animal fodder, soil surface cover, or construction was factored in to determine the mean availability of feedstock for biochar at current. Usage of crop residues as cooking fuel was not considered to compete with biochar production since gasifier systems fulfill both purposes. Farmers who did not indicate any of the predetermined residue uses, confirmed that they burned or dumped the residues instead. In parallel to the diverse usage census, we included two notional scenarios of competitive usages, i.e., low, 20% of straw and non‐straw diverted; and high, 80% of straw and 50% of non‐straw diverted.

**Table 1:** Overview of food crops grown by farmers in the study area

Crop	Respondents (%)
Cassava	100
Maize	97
Bean	82
Groundnut	82
Sorghum	74
Sweet potato	70
Soybean	60
Millet	57
Banana	49
Rice	40
Cowpea	22
Simsim	18
Cotton	5

*Note:* Percentages are based on the census of 60 individuals.

### Sampling of crops

Crop height, stand density, and yield components were measured from 12 farmer fields distributed across the study area to represent the range of soil properties and input management. This sampling was carried out when crops had reached full maturity, taking place from May to July 2016 and 2017. In the first season, sampling was incomplete because part of farmers harvested prematurely due to severe drought (Appendix S1: Table S1). Measurements for groundnut, millet, and rice crops were taken from four quadrats of 1 m^2^ for each farmer field, while, for maize and sorghum, two quadrats of 4 m^2^ were assessed per field. Within each quadrat, the plant density (PD) was determined, and the height (*H*) measured for four plants for maize and sorghum, and six plants for millet, rice, and groundnuts. Measurements of plant height were taken from the soil surface up to the base of the tassel for maize, the base of the panicle for millet, sorghum, and rice, and the last terminal leaf for groundnut. The total fresh mass of straw and panicles or pods of rice, millet, and groundnut were determined for each quadrat, and representative subsamples taken for assessing water content. For maize and sorghum crops, biomass samples were collected from four plants in each quadrat, and the fresh mass of its straw and cobs or panicles was measured. All biomass samples were oven‐dried at 60°C until reaching steady dry mass (DM). Subsequently, the grain of crops was separated from shanks, chaff (i.e., husks and panicle axis) or pods, and the dry mass of individual fractions was measured. The productivity of grain and residues from rice, millet, and groundnut was computed for individual quadrats in farmer fields by multiplying the total fresh mass with the ratio of the dry to fresh mass of the subsample. The productivity of maize and sorghum, on the other hand, was calculated by multiplying the average mass of one plant from the subsample with the plant count.

### Allometric modelling of crop residue yield

Relationships of the total productivity of straw and non‐straw biomass with grain yields of crops, as well as plant height and density (Appendix S1: Table S2), were modeled based on linear mixed effect regressions. Data from individual sampling quadrats were used in developing and testing these prospective quantification models, with the random intercepts of models consisting of specific farmer fields. Sets of data were split into two parts by randomly dividing farmer fields, using one half for generating the models and the other to predict total productivity of crop residues. Measured and predicted residue yields from crops were plotted against each other for cross‐validating the reliability of allometric models. Residual normal distribution and homoscedasticity of mixed effect models were ascertained by plotting residuals against quantiles and fitted values. The total production of straw and non‐straw residues produced by the five studied crops for the whole of Uganda have been calculated using the allometric models based on grain yield drawn from national statistics from 2016 predictions (FAOSTAT 2018). Minimum and maximum values of total residue production were derived from the cross‐validated models.

### Conversion of biomass to biochar and fixed carbon

Information about the mass conversion of crop residues to biochar and the proportion of fixed carbon in biochar from different types of pyrolysis systems was retrieved from publications of Thomson index journals (Appendix S1: Table S3). These calculations were standardized among crop residues by using the conversion rates and fixed carbon contents recorded at a temperature of 500°C, which falls within the optimal range of pyrolysis conditions. If there were multiple reports of conversion factors for a particular crop residue in literature, we calculated and applied a mean of those values. There was a paucity of peer‐reviewed information for chaff from sorghum, and instead, the biochar yield and fixed carbon of its straw was used, whereas conversion factors for chaff and straw of millet were substituted by those from rice. Biochar yields were computed by multiplying the amounts of available crop residues with the proportional mass conversion of biomass to biochar. And, these were further multiplied with the percentage fixed carbon in biochars to calculate the amount of C that is being generated.

### Biochar C sequestration in soil

The accumulation of C in croplands, achieved through annual biochar inputs from residues of major cropping systems over a period of 50 yr, was simulated by a kinetic model including a labile and stable pool with constant rates of mineralization (Foereid et al. 2011). The amount of biochar C that can be locked in soils under a particular scenario is described by the following equation:

$$\begin{array}{cc} \mathrm{BC}S_{t} & =\sum_{t=0}^{n}\left(\mathrm{iB}C_{t}*f+\;\mathrm{labB}C_{t}\right)*\;e^{-k_{1}}\;\\ & \;+\;\left(\mathrm{iB}C_{t}*\left(1-f\right)+\;\mathrm{recB}C_{t}\right)*e^{-k_{2}} \end{array}$$

where BCS is the amount of biochar carbon in soil at time *t* (Mg C/ha), iBC is the input of biochar carbon at time *t* (Mg C·ha^−1^·yr^−1^), *f* is the ratio of labile C in biochar, labBC and recBC are the labile and recalcitrant pool of biochar C in the soil at time *t* (Mg C/ha), *k*_1_ is the fraction of the labile carbon pool that is mineralized (per year), and *k*_2_ is the fraction of stable carbon pool that is mineralized (per year). Orthogonal crosses with maximum and minimum values of each factor were constructed, based on information outlined in this paper, to demonstrate the limits and variance in the potential for locking away C in soils through biochar. The labile carbon fraction of biochar was, respectively, taken as 3–8%, and with a decomposition rate of 90% per year, whereas loss of C from the stable pool was taken as 2–6% per year. Annual inputs of biochar from crop residues, as well as the labile to stable ratio and decomposition rates, under each of the scenarios were kept constant over time, which possibly leads to conservative estimates. This C balance model does not explicitly account for erosive loss of biochar from soils and hence may overestimate the amounts of C retained at a specific farmer fields. Notwithstanding, the upper limit of coefficient *k*_2_ that was used in simulations has been derived from an experiment where lateral transport contributed to the disappearance of biochar (Katterer et al. 2019).

## Results and Discussion

### Yields and usages of crop residues in farming systems of eastern Uganda

**Figure 2:**
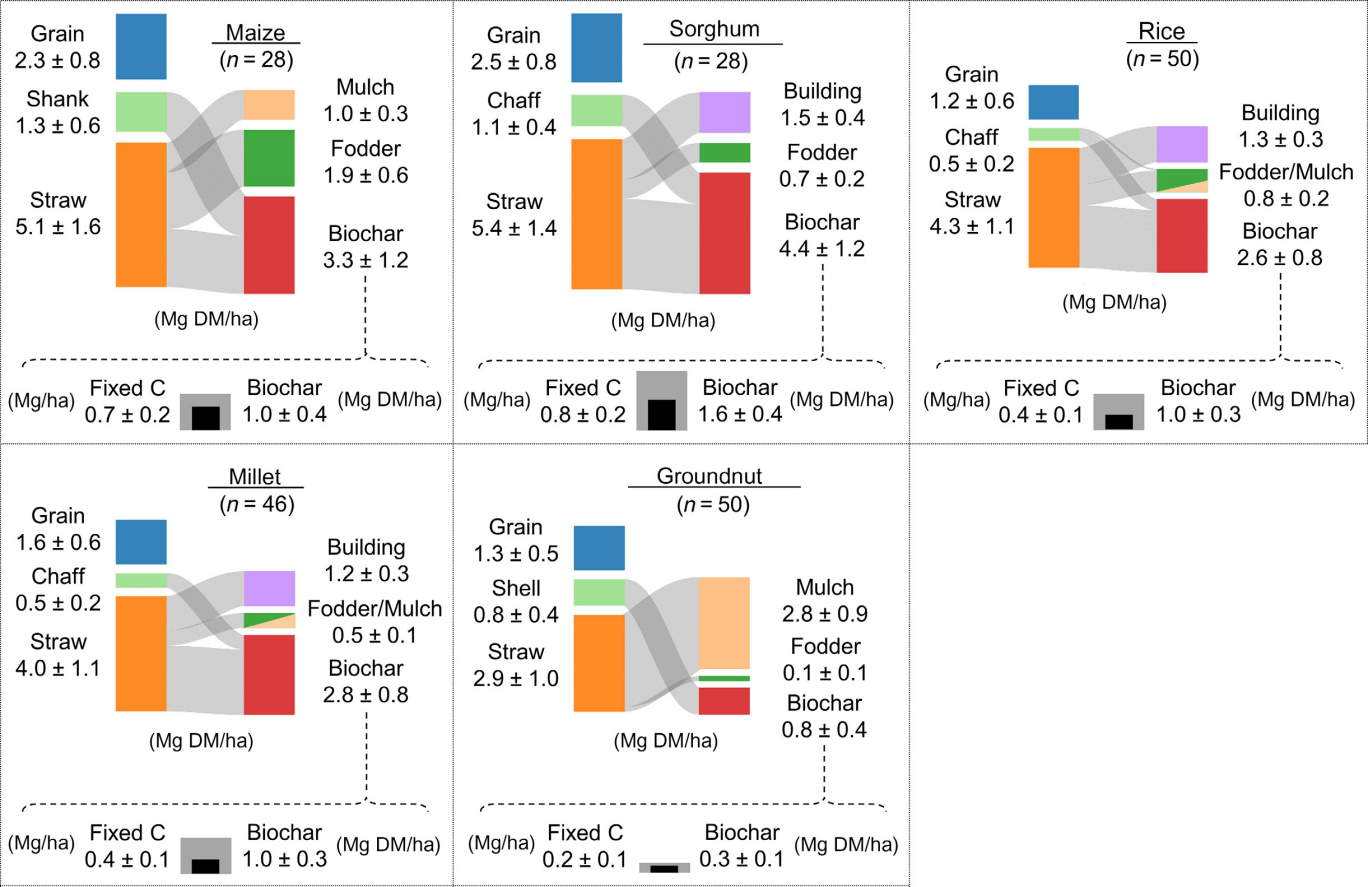
Biomass productivity and flows of residues for staple crops measured in farming systems of eastern Uganda. On the left of each Sankey diagram is yields of straw and non‐straw fractions, and on the right is the allocation of biomass from crops and availability for biochar determined through the household census. The slices in bar chart reflect the proportion of residues going to a particular usage. At the bottom of each graph are the potential rates of biochar and C amendment to soils that were quantified based on mean conversion factors from peer‐reviewed literature. Values are means and standard deviations from sampling quadrats. DM, dry mass.

The five studied crops are an intrinsic part of bi‐modal cropping systems in mid‐altitude regions of Uganda, and of major importance to food production. Grain yields of the crops are generally one‐half or less of reported attainable yields (Fig. 2), which is typical for agroecosystems in SSA, owing to limited access to agricultural inputs and low soil fertility (Sanchez 2010). Straw total biomass yields for maize and sorghum were significantly higher than those of other crops by 0.84–2.47 Mg DM/ha, on average. Rice and millet generated a significant larger amount of straw compared to groundnut, with mean yields amounting to 1.10–1.35 Mg DM/ha more. The quantity of shanks from the maize cobs and chaff from sorghum panicles, in turn, was significantly higher than the yield of non‐straw residues from the other crops by 0.32–0.90 Mg DM/ha, on average. Mean productivity of shells by groundnut was significantly higher compared to the quantity of chaff generated by rice and millet, respectively, yielding 0.31–0.35 Mg DM/ha more. Mean straw yields of the five crops in each of the farmer fields and growing seasons varied by 18–28%, whereas the productivity of non‐straw residues had a relative standard deviation of 25–38%. These findings demonstrate there can be substantial differences in the total productivity of the staple crop biomass waste at the level of individual farm enterprises, and therefore differences in their availability for biochar and potential C sequestration.

The proportion of farmers that reported using straw residues for animal fodder, building material, and mulching ranged from 61% for maize crops to 8% for groundnut crops, while amounting to 12% for rice husks, 5% for groundnut shells, and zero for maize shanks and sorghum and millet chaff (Table 2). Among the interviewed, 92–98% said their household relied on straw and shanks of maize as fuel for cooking energy using conventional incineration‐based stoves, and 10% indicated using sorghum straw or groundnut shells as fuel. Based on these measured usages, straw and chaff from sorghum, on average, provided the largest amount of available biomass among the studied crops, between 1.1–1.8 Mg DM/ha more than residues from maize, rice, and millet (Fig. 2). In its turn, the amount of available maize straw and shanks was significantly greater than that of residues from rice and millet. The availability of residues from groundnut for biochar was the lowest of all crops, this because straw biomass (with C:N ratio <20) does not make an appropriate feedstock for pyrolysis feedstock as it is better directly incorporated into soils or composted.

**Table 2:** Allocation of crop residues to major types of use purposes by farmers in the study area

Crop and residue	Usage (respondents %)				Available for biochar† (biomass %)
	AF	CN	MI	CF	
Maize					
Straw	38	3	20	92	39
Shank	0	0	0	98	100
Sorghum					
Straw	13	27	0	10	60
Chaff	0	0	0	0	100
Rice					
Straw	13	30	5	0	52
Husk	12	0	0	0	88
Millet					
Straw	5	30	8	0	57
Chaff	0	0	0	0	100
Groundnut					
Straw	5	0	3	0	0
Shell	0	0	5	10	95

*Notes:* Percentages are based on the census of 60 individuals. AF, animal fodder; CF, cooking fuel; CN, construction; MI, mulching or incorporation.

† Difference with sum of AF, CN, and MI.

These findings indicate that substantial amounts of biomass wastes from staple foods are left unused in the studied farming systems and, thus, potentially available for biochar. The apparently low usage of residues is a consequence of the low number of small livestock owned by farmers (Appendix S1: Table S4); this is illustrated by the mounds of rice husks piling around the mills, which could theoretically be fed to poultry or swine. Admittedly, the limited usage of residues for mulching soils in the studied farming systems is ascribed to the low adoption rates of conservation agriculture in Africa (Corbeels et al. 2014). Many farmers in Uganda, and SSA, still incinerate crop residues in the field, easing land cultivation and utilizing the ashes as fertilizer or liming agent. Pyrolyzing biomass wastes from staple crops using gasifier energy appliances that generate biochar can offer a more profitable alternative over incineration for farmers in Level 1 and 2 economies.

### Forecasting of crop residue yields

**Figure 3:**
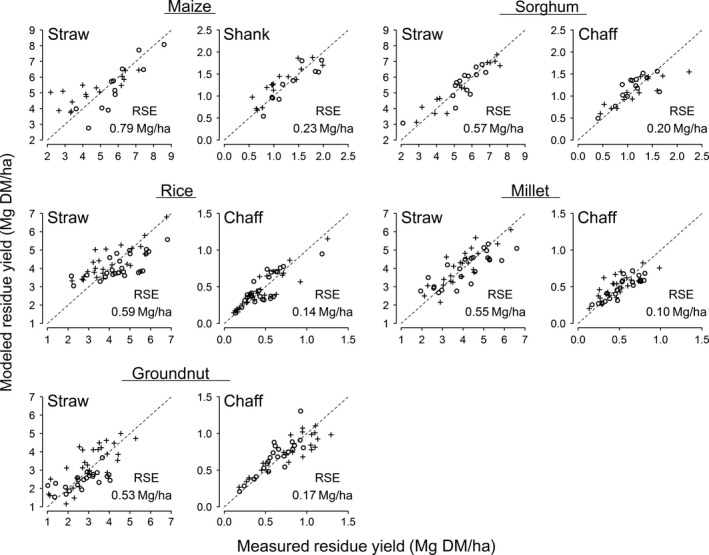
Models for prospective quantification of crop biomass yields using grain productivity records. Each plot is showing the goodness of fit between measured and predicted productivity of crop residues. Different symbols display the two subsets of data used for either model development or validation. RSE, residual standard error.

Cross‐validation of residue yield predictions based on grain yields demonstrated moderate to good agreement with measured values for each crop across large yield ranges despite the relatively small sample size (Fig. 3). The mean errors on quantifying straw yields based on that of grain amounted to 0.53–0.79 Mg DM/ha for the five crops. For sorghum and rice, the models tend to overestimate measured values at low levels of straw productivity, but otherwise, errors were evenly distributed in this study. Mean errors on non‐straw yields quantified based on grain productivity amounted to 0.08–0.23 Mg DM/ha for the five crops. For millet chaff, the model tends to underestimate measured values at high levels of productivity, but otherwise, errors were again evenly distributed in this study. These results demonstrate that the amounts of individual residues produced by the five crops can be reliably forecasted through linear mixed‐effect modeling of economic grain yields. Moreover, errors on the prospective quantification of crop biomass wastes stand to be reduced if larger sample sizes are taken than in this study. Applications of our residue forecasting models based on food production data notably extend from quadrat sampling to farmer fields, as well as upscaling of national agricultural statistics.

Cross‐validation of residue yield predictions according to the density and height of crop stands exhibited poor agreement with actual values for all investigated (Appendix S1: Fig. S1), with mean errors being up to 2.4 times greater than for models based on grain yields. Deviances of residuals for prospective quantification of straw productivity using plant height and density amounted to 0.67–1.31 Mg DM/ha for all investigated crops, whereas errors on non‐straw yields measured 0.12–0.36 Mg DM/ha. For sorghum straw, and all residue fractions of rice and millet, these allometric models gravely under‐ or overestimated actual values, whereas errors on quantifying other crop residues were evenly distributed across the sampled productivity ranges. The poor goodness of fit achieved by this approach can be ascribed to interactive effects of genetic, agricultural, and environmental factors on relationships between plant density and crop growth (Friedman 2016). Forecasting crop residue yields through this approach may be improved by taking larger sample sizes and including more covariates in the models that influence relationships.

### Potential C sequestration in soils with crop residue biochar

**Figure 4:**
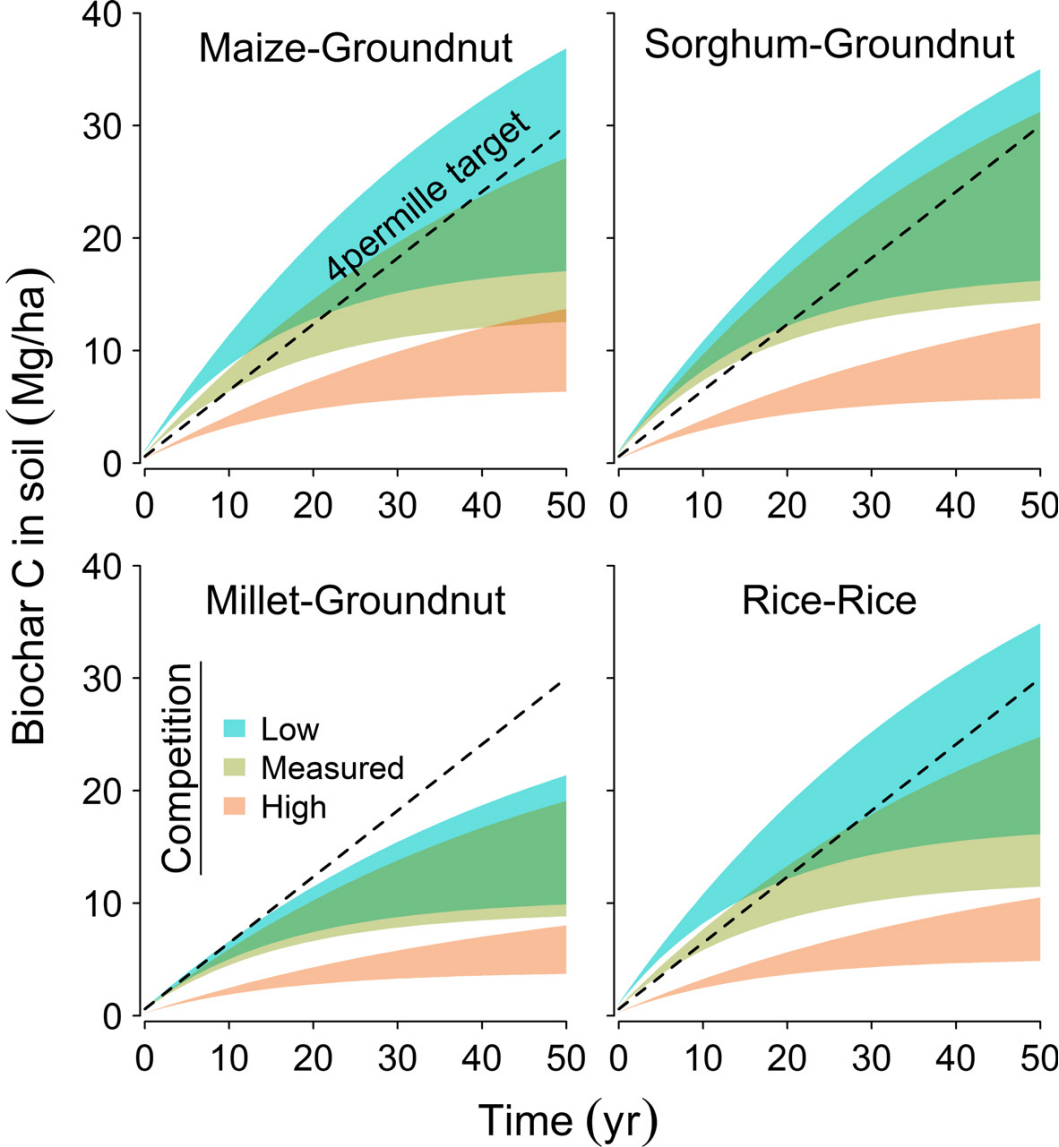
Simulated C sequestration in soils of major cropping systems that receive “circular” amendment of biochar from residues. Envelope curves illustrate different scenarios of competitive usage for crop residues; i.e., low, 20% of straw and non‐straw diverted; measured, allocation indicated by census; and high, 80% of straw and 50% of non‐straw diverted. The boundaries of polygons are set by decomposition factors of biochar in peer‐reviewed literature, i.e., upper, *f* = 3%, *k*_2_ = 2% per year; lower, *f* = 8%, *k*_2_ = 6% per year (f is the ratio of labile C in biochar and k_2_ is the fraction of the stable carbon pool that is mineralized every year). Diagonal lines show a constant increase in soil C stocks of 0.6 Mg·ha^−1^·yr^−1^, the “4 per mille” target.

Accumulation of biochar C in soils, simulated based on average yields of crop residues measured in farmer fields, exhibited notable differences between the investigated cropping systems and degrees of competitive resource usage (Fig. 4). Apparent rates of C build‐up in farmlands are exponentially diminishing because mineralization coefficients for biochar amendments made over time have been kept constant in this study. Recycling of crop residues as biochar for 50 yr, sequestered an average of 6–27 Mg C/ha in the various cropping systems and scenarios of competitive usage, with a deviation of 4–20 Mg C/ha between simulations with low and high biochar decomposition factors. Based on the allocation of residues measured by the census, maize–groundnut and sorghum–groundnut rotations and rice paddies could sustain biochar C sequestration rates in soils of 0.60–0.97 Mg·ha^−1^·yr^−1^ for 3–23 yr. Residues from millet–groundnut rotations, under the measured availability for biochar, could increase soil C stocks by 0.50–0.60 Mg·ha^−1^·yr^−1^ for 3–8 yr. When competitive usage of crop residues is high, i.e., 80% for straw and 50% for non‐straw biomass, the potential rates of biochar C sequestration in each of the four cropping systems are between 0.20 and 0.43 Mg·ha^−1^·yr^−1^ for a period of 2–25 yr. If competitive usage of biomass wastes is low, i.e., 20% for straw and non‐straw residues, then maize–groundnut and sorghum–groundnut rotations and rice paddies could sustain biochar C sequestration rates of 0.6–1.15 Mg·ha^−1^·yr^−1^ for a period of 8–31 yr. Biochar derived from residues of millet–groundnut rotations could increase soil C stocks by 0.50–0.67 Mg·ha^−1^·yr^−1^ for 4–14 yr when resource competition is low. These particular findings illustrate that recycling staple crop residues as biochar in eastern Uganda could substantially contribute to sequestering C in soils, and may even exceed the ambitious target set by the “4 per mille” strategy (Minasny et al. 2017).

National‐scale production of straw and non‐straw residues by the five crops in Uganda, forecasted based on economic grain yield statistics, could generate a total of 0.8–1.0 Tg biochar C annually, under levels of competitive usages measured by the census (Table 3). If there is high competitive usage of residues, then ~0.3 Tg biochar C could be produced countrywide each year, whereas 1.1–1.3 Tg biochar C could be produced if allocation of biomass to fodder, construction, and mulching is low. The total amount of C that could potentially be added to Ugandan soils through biochar from the five crops investigated is, respectively, equal to 0.5–2% of the global per capita footprint of fossil fuel and cement GHG emissions recorded in 2014, or 19–77% of the actual carbon footprint in Uganda (World Bank 2018). At global scale, it is estimated that approximately 2% of anthropogenic GHG emissions could be abated through biochar C sequestration in soils under maximum realistic scenarios (Griscom et al. 2017). The mitigation potential of biochar from unused residues of maize, sorghum, rice, millet and groundnut in Uganda alone, is remarkably close to this value demonstrating the potential of this simple climate solution. Widespread cultivation of the five investigated crops as staple food and bimodal cropping seasons in the country are making it possible to achieve such high rates of soil C sequestration with their biomass residues. Our assessment consequently indicates that circular production and soil amendment of biochar derived from unused crop residues and general biomass wastes may represent an effective strategy for offsetting GHG emissions in Uganda.

**Table 3:** Total annual residue production from the five studied crops in Uganda forecasted using models based on grain yields

Crop	Production (Gg DM)			Amount of biochar C (Gg DM) competitive residue usage		
	Grain†	Straw	Shank, Chaff, or Shell	Low	Measured	High
Maize	2,663					
Minimum		3,990	1,652	928	678	232
Maximum		4,542	1,853	1,051	765	263
Sorghum	315					
Minimum		431	116	82.5	70.7	20.6
Maximum		539	155	105	90.3	26.2
Rice	247					
Minimum		302	99.2	51.5	41.0	12.9
Maximum		355	104	58.4	45.9	14.6
Millet	234					
Minimum		335	56.1	48.0	39.3	12.0
Maximum		417	56.4	57.5	46.1	14.4
Groundnut	85					
Minimum		121	54.4	10.2	12.7	2.5
Maximum		149	68.3	12.8	15.9	3.2
Total	3,544					
Minimum		5,179	1,978	1,120	841	280
Maximum		6,002	2,237	1,284	963	321

*Note:* The minimum and maximum production of residues straw, shank, chaff, shell) by crops are determined through cross‐validation of models. Amounts of C in crop residue derived biochar that can be generated nationwide are calculated for different scenarios of competitive usages, i.e., low, 20% of straw and non‐straw diverted; measured, allocation indicated by census; and high, 80% of straw and 50% of non‐straw diverted. DM, dry mass.

† Data for 2016 retrieved from http://www.fao.org/faostat

### Co‐benefits for agriculture and energy

Average measured biomass yields from rotations of maize, sorghum or millet with groundnut lead to potential soil biochar amendments of 0.74–2.1 Mg DM·ha^−1^·yr^−1^, and 0.80–2.7 Mg DM·ha^−1^·yr^−1^ for rice paddies, under the various competitive use scenarios. Comparable application rates have been shown to result in immediate gains in crop productivity since input of biochar generally exhibits high efficacy at low rates (Liu et al. 2013). Moreover, long‐lasting increases of crop yields that are demonstrated in tropical agroecosystems permit the gradual building up of stocks in soils when availability of residues is low, as is the case for majority of farmers in SSA. The national‐scale production of biomass wastes from the five crops in Uganda forecasted based on grain yield statistics illustrates this is a viable transformation pathway leading to soil biochar application and fulfills the remit of sustainable agricultural intensification. Increasing the productivity of crops through soil biochar amendment would make more biomass resources available to farmers further improving the fertility of croplands and sequestering C. However, recommendations about effective biochar dosages and co‐application of inputs under heterogeneous conditions in Uganda need to be developed further for optimization.

In addition to generating biochar, the gasification of residues from crops also produces synthesized combustible gasses and heat that can power various energy appliances of rural households and enterprises. Based on average biomass availability in the study area under low to high competitive usage, and gross calorific values of residues from literature ranging between 12.6 and 17.7 MJ/kg, we calculate that the potential energy yield from croplands under rotation of maize, sorghum or millet with groundnut and mono‐crop rice is amounting to 10–51 GJ·ha^−1^·yr^−1^, at a modest conversion efficiency of 50%. Recent surveys in rural households of East Africa suggest mean annual rates of firewood consumption of 440–640 kg per capita (Jung and Huxham 2018, Stoppok et al. 2018). Assuming a gross calorific value of 18.5 MJ/kg for wood biomass, our assessment shows that 0.2–1.2 ha of land is needed to acquire sufficient residues from the one of the four investigated cropping systems to substitute the firewood requirement of one person. At a national scale, the potential energy yield from residues of maize, sorghum, rice, millet and groundnut crops under the scenarios of low to high competitive usage, amounts to 12–49 PJ/yr, going by the above gross calorific values and again 50% conversion efficiency. Based on a gross energy balance, this means that available biomass wastes in Uganda from just the five investigated crops could replace wood fuels consumed by 1.0–6.3 million people in rural communities.

This novel assessment reveals that substantial contributions to mitigating GHG emissions can be made by sequestering C through soil biochar amendments from residues in cereal–legume food systems of eastern Uganda, and elsewhere in the country, despite biomass usage for other purposes. Findings from our study also demonstrate major benefits for agricultural and energy production by diverting available biomass wastes from the investigated staple crops to gasification appliances. The framework we developed for prospective quantification of residue availabilities based their relationships with grain yield and census of competitive usage can be applied elsewhere, if robustly validated and could underpin carbon economy based development specifically in Level 1 economic areas, characterized by high levels of subsistence farming and biomass fuel use. With the help of these tools, it is possible to make a rapid assessment of the biophysical potential of crop residues for amending biochar to soils and generating energy at the level of individual farmer fields up to national scale.

## Data Availability

Data of crop biomass yield measurements carried out by this study are available in tabular text files via CKAN repositories as follows: maize, https://doi.org/10.25502/z5dp-be17/d ; sorghum, https://doi.org/10.25502/fbgw-1m42/d; rice, https://doi.org/10.25502/cne2-h823/d; millet, https://doi.org/10.25502/edk6-ac73/d; and groundnut, https://doi.org/10.25502/eerp-3f45/d. Each has the R scripts that were used for cross‐validating linear mixed models to predict residue yields based on grain productivity, and plant density, and height.

## References

[eap1984-bib-0001] Amundson, R. , and L. Biardeau . 2018 Soil carbon sequestration is an elusive climate mitigation tool. Proceedings of the National Academy of Sciences USA 115:11652–11656. 10.1073/pnas.1815901115 PMC6243274 30425181

[eap1984-bib-0002] Bach, M. , B. Wilske , and L. Breuer . 2016 Current economic obstacles to biochar use in agriculture and climate change mitigation. Carbon Management 7:183–190.

[eap1984-bib-0003] Blanco‐Canqui, H. 2017 Biochar and soil physical properties. Soil Science Society of America Journal 81:687–711.

[eap1984-bib-0004] Corbeels, M. , et al. 2014 Understanding the impact and adoption of conservation agriculture in Africa: a multi‐scale analysis. Agriculture, Ecosystems and Environment 187:155–170.

[eap1984-bib-0005] de la Rosa, J. M. , M. Rosado , M. Paneque , A. Z. Miller , and H. Knicker . 2018 Effects of aging under field conditions on biochar structure and composition: implications for biochar stability in soils. Science of the Total Environment 613–614:969–976. 10.1016/j.scitotenv.2017.09.124 28946384

[eap1984-bib-0006] FAOSTAT . 2018 Food and Agriculture Organization of the United Nations Statistics Database. FAO, Rome, Italy.

[eap1984-bib-0007] Foereid, B. , J. Lehmann , and J. Major . 2011 Modeling black carbon degradation and movement in soil. Plant and Soil 345:223–236.

[eap1984-bib-0008] Friedman, S. P. 2016 Evaluating the role of water availability in determining the yield‐plant population density relationship. Soil Science Society of America Journal 80:563–578.

[eap1984-bib-0011] Griscom, B. W. , et al. 2017 Natural climate solutions. PNAS 114:11645–11650. 29078344 10.1073/pnas.1710465114 PMC5676916

[eap1984-bib-0009] Häring, V. , D. Manka'abusi , E. K. Akoto‐Danso , S. Werner S , K. Atiah , C. Steiner , D. J. Lompo , S. Adiku , A. Buerkert , and B. Marschner . 2017 Effects of biochar, waste water irrigation and fertilization on soil properties in West African urban agriculture. Scientific Reports 7:10738. 28878251 10.1038/s41598-017-10718-y PMC5587607

[eap1984-bib-0010] Hood‐Nowotny, R. , A. Watzinger , A. Wawra , and G. Soja . 2018 The impact of biochar incorporation on inorganic nitrogen fertilizer plant uptake; an opportunity for carbon sequestration in temperate agriculture. Geosciences 8:420.

[eap1984-bib-0012] Jeffery, S. , D. Abalos , M. Prodana , A. C. Bastos , J. W. van Groeningen , B. A. Hungate , and F. Verheijen . 2017 Biochar boosts tropical but not temperate crop yields. Environmental Research Letters 12:053001.

[eap1984-bib-0013] Jung, J. , and M. Huxham . 2018 Firewood usage and indoor air pollution from traditional cooking fires in Gazi Bay, Kenya. BioScience Horizons 11:1–12.

[eap1984-bib-0014] Katterer, T. , D. Roobroeck , O. Andren , G. Kimutai , E. Karltun , H. Kirchmann , G. Nyberg , B. Vanlauwe , and de Roing Nowina K. . 2019 Biochar addition persistently increased soil fertility and yields in maize‐soybean rotations over 10 years in sub‐humid regions of Kenya. Field Crops Research (Submitted for 2nd round of revisions, 15 February 2019)

[eap1984-bib-0015] Kimetu, J. M. , and J. Lehmann . 2010 Stability and stabilization of biochar and green manure in soil with different organic carbon contents. Australian Journal of Soil Research 48:577–585.

[eap1984-bib-0016] Liang, B. , et al. 2006 Black carbon increases cation exchange capacity in soils. Soil Science Society of America Journal 70:1719–1730.

[eap1984-bib-0017] Liu, X. , A. Zhang , C. Ji , S. Joseph , R. Bian , L. Li , G. Pan , and J. Paz‐Ferreiro . 2013 Biochar's effect on crop productivity and the dependence on experimental conditions—a meta‐analysis of literature data. Plant and Soil 373:583–594.

[eap1984-bib-0018] Minasny, B. , et al. 2017 Soil carbon 4 per mille. Geoderma 292:59–86.

[eap1984-bib-0019] Sanchez, P. 2010 Tripling crop yields in tropical Africa. Nature Geoscience 3:299–300.

[eap1984-bib-0020] Sanford, L. , and J. Burney . 2015 Cookstoves illustrate the need for a comprehensive carbon market. Environmental Research Letters 10:084026.

[eap1984-bib-0021] Schlesinger, W. H. , and R. Amundson . 2018 Managing for soil carbon sequestration: let's get realistic. Global Change Biology 25:1–4. 30485613 10.1111/gcb.14478

[eap1984-bib-0022] Smith, P. 2016 Soil carbon sequestration and biochar as negative emission technologies. Global Change Biology 22:1315–1324. 26732128 10.1111/gcb.13178

[eap1984-bib-0023] Stoppok, M. , A. Jess , R. Freitag , and E. Alber . 2018 Of culture, consumption and cost: a comparative analysis of household energy consumption in Kenya, Germany and Spain. Energy Research and Social Science 40:127–139.

[eap1984-bib-0024] Wang, J. , Z. Xiong , and Y. Kuzyakov . 2016 Biochar stability in soil: meta‐analysis of decomposition and priming effects. GCB Bioenergy 8:512–523.

[eap1984-bib-0025] World Bank 2018 CO_2_ emissions (metric tons per capita). Washington, D.C. https://data.worldbank.org/indicator/EN.ATM.CO2E.PC

