# Supplementary material for: Biophysical potential of crop residues for biochar carbon sequestration, and co‐benefits, in Uganda
Source: Ecol Appl. 2019 Aug 30;29(8):e01984. doi: 10.1002/eap.1984 (PMC6916656; doi:10.1002/eap.1984)

Supporting Information. Dries Roobroeck, Rebecca Hood-Nowotny, Dianah Nakubulwa, John-Baptist Tumuhairwe, Jackso[illegible]ajaliwa, Isaac Ndawula, and Bernard Vanlauwe. 2019. Biophysical ~~potentials~~ of crop residues for biochar carbon sequestration, and co-benefits, in Uganda. *Ecological Applications.*

Appendix S1

Table S1. Characteristics of soils and weather in the studied farming systems. Soil properties are determined from 34 farmer fields in the same area where agronomic trials were carried out. Cumulative rainfall and mean temperatures during the two growing seasons of biomass sampling.

	Soil depth (cm)		Season (Feb–Jun)	
	0–15	15–30	2016	2017
Soil characteristics				
Sand (%)[1]	63.0 ± 7.1	60.6 ± 6.3		
Clay (%)[1]	25.5 ± 6.2	29.0 ± 6.5		
Silt (%)[1]	11.6 ± 3.8	10.5 ± 7.8		
pH_{water}[2]	5.94 ± 0.45	5.86 ± 0.55		
extractable P (mg kg^{-1})[3]	6.78 ± 6.96	4.95 ± 7.92		
Weather characteristics				
Cumulative rainfall (mm)[4]			682 ± 39	747 ± 31
Mean air temperature (°C)[5]			26.5	24.8

[1]measured by conventional sedimentation method; [2]from 1:2 soil and distilled water w/v solution and measured with electrode; [3]from 1:2 soil and 0.01M sodium bicarbonate w/v mixture and measured colorimetrically; [4]retrieved from 0.05 x 0.05 degree raster data from the Climate Hazards Group Infrared Precipitation (CHIRPS) with Station data; [5]from Uganda National Meteorological Authority.

Table S2. Allometric characteristics of crops investigated by this study. Values are mean, minimum and maximum from all sampled quadrats.

Crop	Height (m)			Plant density (counts m^{-2})		
	Min	Max	Mean	Min	Max	Mean
Groundnut	0.34	0.75	0.51	8	44	22
Maize	1.13	2.58	1.90	3	9	6
Millet	0.39	1.15	0.84	30	164	83
Rice	0.54	1.45	1.00	10	46	24
Sorghum	1.20	2.40	1.77	6	27	13

Table S3. Conversion factors for pyrolysis of crop residues from peer-reviewed literature. Proportions of biochar yield and fixed C are respectively based on the dry weight of feedstocks before and after pyrolysis.

Crop residue	Biochar (%)	Fixed (%)	Reference
Maize straw	38.09	37.97	Morales et al. 2015 (1)
	28.89	62.26	Enders et al. 2012 (2)
	30.9	76.6	Liu et al. 2014 (3)
	32.29	62.9	Capunitan & Capareda 2012 (4)
Maize cob	28.8	89.9	Shariff et al. 2016 (5)
	23.3	81.6	Liu et al. 2014 (3)
Sorghum straw	36.9	51	Santos & Capareda 2016 (6)
Rice husks	38.04	55.3	Cheng & Wang 2017 (7)
	47.49	47.09	Zhang et al. 2017 (8)
	47.83	38.5	Crombie et al. 2013 (9)
Rice straw	41	39.1	Lee et al. 2014 (10)
	39	44.9	Wu et al. 2012 (11)
	25.4	38.72	Park et al. 2014 (12)
Groundnut hulls	32	72.9	Zhao et al. 2013 (13)

Digital Object Identifier: (1) 10.1016/j.biortech.2015.02.043 ;
(2) 10.1016/j.biortech.2012.03.022 ; (3) 10.1016/j.biortech.2014.07.077;
(4) 10.1016/j.fuel.2011.12.029 ; (5) 1999.2/10005789 ; (6) 10.1007/s13399-015-0191-5 ;
(7) 10.3390/en10111814 ; (8) 10.15376/biores.12.3.4652-4669 ; (9) 10.1111/gcbb.12030 ;
(10) 10.1016/j.biortech.2013.08.135 ; (11) 10.1016/j.biombioe.2012.09.034 ;
(12) 10.1016/j.biortech.2013.12.084 ; (13) 10.1016/j.jhazmat.2013.04.015

Table S4. Quantification of livestock numbers in the study area. Proportions of ownership and mean density are based on the census of sixty farmers. Numbers of different livestock in households were standardized using the tropical unit scalar, and the mean density is calculated only for farmers that own livestock.

Types of livestock	Ownership (% respondents)	Density (TLU hh^{-1})*
Cow	60	2.41
Oxen	18	1.4
Goat/Sheep	70	0.32
Chicken/Turkey	77	0.12
Pig	27	0.59

*Tropical livestock units per household

Figure S1. Models for prospective quantification of crop biomass yields based on plant height and stand. Panel plot showing the goodness of fit of measured and predicted productivity of crop residues. Different symbols represent the two subset of data used for either model development or validation. RSE: residual standard error.

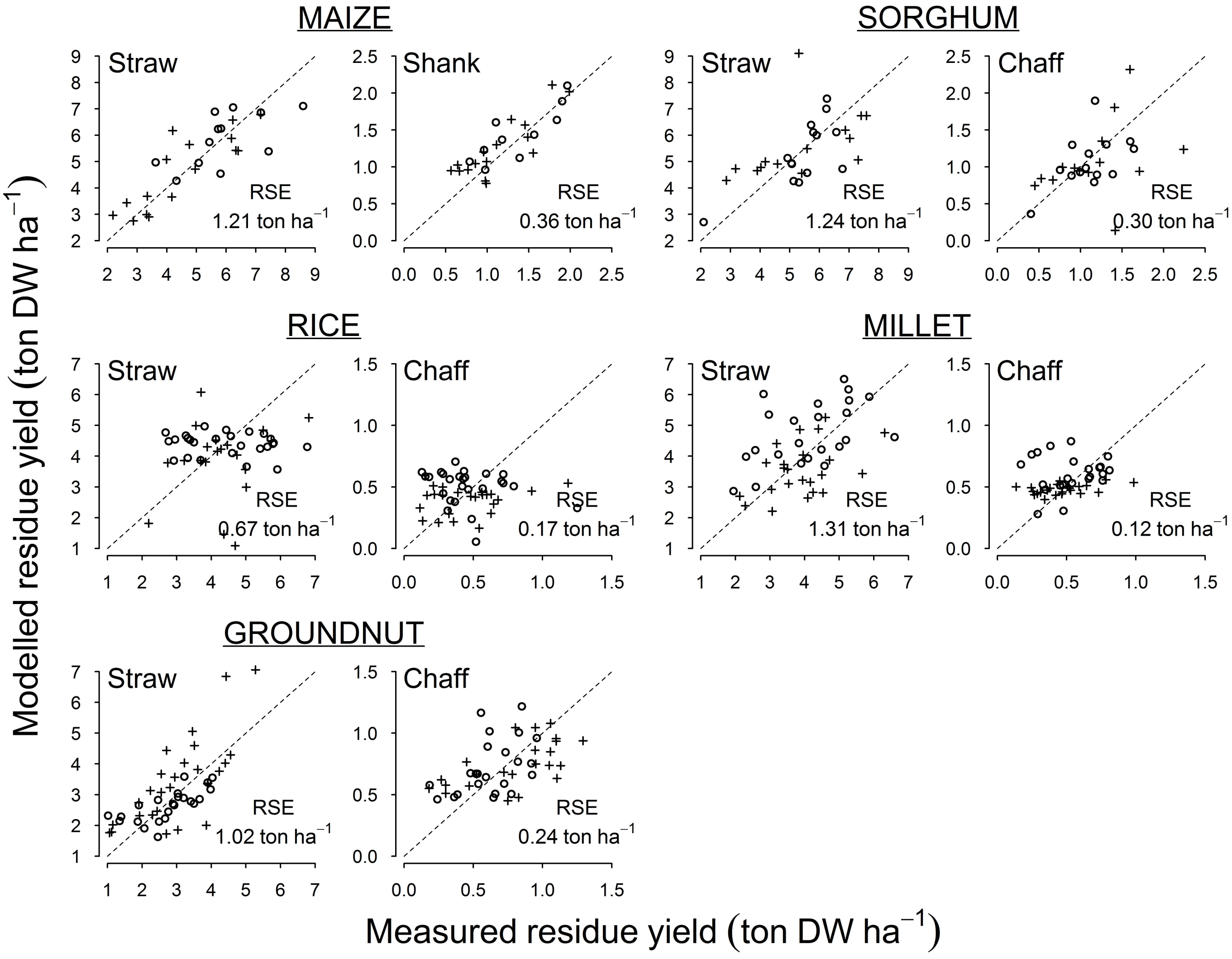

Supplement: Supplementary file 1 [file EAP-29-na-s001.pdf]
